# Pregnancy as an opportunity to prevent type 2 diabetes mellitus: FIGO Best Practice Advice

**DOI:** 10.1002/ijgo.14537

**Published:** 2023-01-12

**Authors:** Sumaiya Adam, Harold David McIntyre, Kit Ying Tsoi, Anil Kapur, Ronald C. Ma, Stephanie Dias, Pius Okong, Moshe Hod, Liona C. Poon, Graeme N. Smith, Lina Bergman, Esraa Algurjia, Patrick O'Brien, Virna P. Medina, Cynthia V. Maxwell, Lesley Regan, Mary L. Rosser, Bo Jacobsson, Mark A. Hanson, Sharleen L. O'Reilly, Fionnuala M. McAuliffe, the FIGO Committee on the Impact of Pregnancy on Long‐term Health and the FIGO Division of Maternal and Newborn Health

**Affiliations:** ^1^ Department of Obstetrics and Gynecology, School of Medicine, Faculty of Health Sciences University of Pretoria Pretoria South Africa; ^2^ Diabetes Research Centre, Faculty of Health Sciences University of Pretoria Pretoria South Africa; ^3^ Mater Health University of Queensland, Mater Health Campus South Brisbane Queensland Australia; ^4^ Department of Medicine and Therapeutics The Chinese University of Hong Kong Hong Kong SAR China; ^5^ World Diabetes Foundation Denmark; ^6^ Hong Kong Institute of Diabetes and Obesity The Chinese University of Hong Kong Hong Kong China; ^7^ Biomedical Research and Innovation Platform (BRIP), South African Medical Research Council Cape Town South Africa; ^8^ Department of Obstetrics and Gynecology St Francis Hospital Nsambya Kampala City Uganda; ^9^ Helen Schneider Hospital for Women Rabin Medical Center Petah Tikva Israel; ^10^ Sackler Faculty of Medicine Tel Aviv University Tel Aviv Israel; ^11^ Department of Obstetrics and Gynecology Prince of Wales Hospital, The Chinese University of Hong Kong Hong Kong China; ^12^ Department of Obstetrics and Gynecology, Kingston Health Sciences Centre Queen's University Kingston Ontario Canada; ^13^ Department of Obstetrics and Gynecology, Institute of Clinical Sciences Sahlgrenska Academy, University of Gothenburg Gothenburg Sweden; ^14^ Department of Obstetrics and Gynecology Stellenbosch University Cape Town South Africa; ^15^ Department of Women's and Children's Health Uppsala University Uppsala Sweden; ^16^ The World Association of Trainees in Obstetrics and Gynecology (WATOG) Paris France; ^17^ Elwya Maternity Hospital Baghdad Iraq; ^18^ Institute for Women's Health University College London London UK; ^19^ Department of Obstetrics and Gynecology, Faculty of Health Universidad del Valle, Clínica Imbanaco Quirón Salud, Universidad Libre Cali Colombia; ^20^ Maternal Fetal Medicine Sinai Health and Women's College Hospital University of Toronto Ontario Canada; ^21^ Imperial College London London UK; ^22^ Department of Obstetrics and Gynecology Columbia University Irving Medical Center New York New York USA; ^23^ Department of Obstetrics and Gynecology Sahlgrenska University Hospital/Ostra Gothenburg Sweden; ^24^ Department of Genetics and Bioinformatics, Domain of Health Data and Digitalisation, Institute of Public Health Oslo Norway; ^25^ Institute of Developmental Sciences University Hospital Southampton Southampton UK; ^26^ NIHR Southampton Biomedical Research Centre University of Southampton Southampton UK; ^27^ UCD Perinatal Research Centre, School of Medicine University College Dublin, National Maternity Hospital Dublin Ireland; ^28^ School of Agriculture and Food Science University College Dublin Dublin Ireland

**Keywords:** cardiometabolic, diabetes, gestational diabetes, health systems, hyperglycemia, prevention

## Abstract

Gestational diabetes (GDM) impacts approximately 17 million pregnancies worldwide. Women with a history of GDM have an 8–10‐fold higher risk of developing type 2 diabetes and a 2‐fold higher risk of developing cardiovascular disease (CVD) compared with women without prior GDM. Although it is possible to prevent and/or delay progression of GDM to type 2 diabetes, this is not widely undertaken. Considering the increasing global rates of type 2 diabetes and CVD in women, it is essential to utilize pregnancy as an opportunity to identify women at risk and initiate preventive intervention. This article reviews existing clinical guidelines for postpartum identification and management of women with previous GDM and identifies key recommendations for the prevention and/or delayed progression to type 2 diabetes for global clinical practice.

## Synopsis

The prevalence of gestational diabetes is increasing. We outline key recommendations for the prevention and/or delayed progression to type 2 diabetes.

## 1 INTRODUCTION

### 1.1 Significance of diabetes in pregnancy and long‐term risks

Globally, it is estimated that around 537 million people are currently living with diabetes, with projections expected to increase to more than 643 million people by 2030.^1^ Pregnancy is associated with multiple structural and functional changes and can be viewed as a biological “stress test” for various maternal organ systems. Growing evidence suggests that pregnancy complications are markers and accelerators of maladaptive maternal physiology, especially for the cardiovascular and metabolic systems. Gestational diabetes mellitus (GDM) is one of the most common complications of pregnancy, characterized by hyperglycemia first recognized during pregnancy that usually resolves immediately postpartum, but still carries long‐term risks.^2^ GDM impacts approximately 13.4% (around 17.0 million) of pregnancies worldwide, with both mother and infant at increased risk of developing type 2 diabetes and other health complications later in life (Table 1).^1^, ^3^, ^4^, ^5^, ^6^

**TABLE 1:** Long‐term complications of gestational diabetes

Complications for women	Complications for the offspring
Hypertension Type 2 diabetes Vascular dysfunction Nonalcoholic fatty liver disease Dyslipidemia Chronic inflammation Chronic kidney disease Ischemic heart disease	Childhood obesity Excess abdominal adiposity Metabolic syndrome Hyperinsulinemia Disordered glucose regulation in adolescents Higher blood pressure Possible early‐onset cardiovascular disease Possible attention deficit hyperactivity disorder and autism spectrum disorders Earlier onset type 2 diabetes Higher risk of GDM in female offspring

Women with a history of GDM have an 8–10‐fold higher risk of developing type 2 diabetes compared with women without a history of GDM, which is highest 3–6 years after a GDM pregnancy.^7^, ^8^, ^9^, ^10^ In addition to an increased risk of type 2 diabetes, a small percentage (0%–9.45%) of women with a history of GDM develop type 1 diabetes postpartum.^11^ Furthermore, women who miss their postnatal follow‐up are also at high risk of developing cardiovascular disease (CVD). A recent meta‐analysis involving over 5 million women showed that women with a history of GDM have a 2‐fold higher risk of developing CVD compared with women without GDM.^12^ This highlights the urgent need for early and ongoing proactive surveillance and effective preventive strategies for type 2 diabetes and CVD.

Women who require pharmacologic treatment, in particular insulin, to control hyperglycemia in pregnancy, are at high risk of type 2 diabetes progression.^13^ Treatment with insulin indicates the inability of beta cell mass to increase insulin secretion in the face of increasing insulin resistance as a consequence of pregnancy‐induced metabolic changes. These women are unable to achieve and maintain required glycemic control by nutrition therapy alone. Accordingly, women treated with insulin in pregnancy have a 3‐fold increased risk of type 2 diabetes progression, compared with women with GDM who did not require insulin treatment.^14^

Pregnancy provides a window that offers a glimpse of forthcoming adverse maternal health conditions.^15^ Understanding this enables heightened awareness, a priori prediction, early detection, and most importantly, an opportunity to implement preventive interventions. A clear pathway for identifying and managing women with a previous history of GDM in the early postnatal period is needed.

Several factors are associated with a more rapid progression to type 2 diabetes. These include: (1) hyperglycemia diagnosed in the first trimester; (2) the degree of glucose intolerance and insulin required during pregnancy; (3) gestational age at diagnosis of GDM; (4) excessive weight gain during pregnancy; (5) inability to shed pregnancy‐induced weight gain postdelivery; (6) history of GDM in an earlier pregnancy; and (7) shorter duration of breastfeeding. In addition, longer periods of postpartum follow‐up to test for type 2 diabetes will increase the rate of diagnosis, thus providing an opportunity for earlier intervention, and hence, prevention of long‐term complications.^5^, ^6^

On the other hand, there is also the need to identify women presenting with GDM who may carry a lower risk or who have specific needs during pregnancy. One such group includes women who have monogenic forms of diabetes or a form of Maturity Onset Diabetes of the Young (MODY), including those harboring mutations in the glucokinase (GCK) gene. It is now appreciated that these women may present as hyperglycemia in pregnancy. For example, in a population‐based Atlantic Diabetes in Pregnancy (Atlantic DIP) study, the prevalence of GCK‐MODY was 1.1 in 1000 (95% CI, 0.3–2.9 in 1000), and the prevalence of GCK‐MODY in GDM was 0.9% (95% CI, 0.3–2.3),^16^, ^17^ although this varied according to different populations. There are certain clinical criteria that can facilitate identification of these women, who may warrant further evaluation to ensure optimal management of hyperglycemia in pregnancy for optimal fetal growth.^18^

### 1.2 Barriers and factors contributing to poor postpartum screening in women with a history of GDM

Despite clear evidence that GDM imposes a substantial risk for women after delivery, postpartum follow‐up remains limited in most parts of the world.^6^, ^10^, ^11^ Poor follow‐up rates in women with GDM are due to both healthcare system and personal factors, as well as patient‐related barriers (Table 2). After delivery, many women experience emotional stress and anxiety as they navigate adjusting to motherhood.^19^, ^20^ In such situations, women find it difficult to attend postpartum testing, especially in a fasting state. Research indicates that women who do return for postpartum follow‐up experience lack of continuity and improper care and coordination from the health system.^21^, ^22^, ^23^ Nonpatient‐centric care often occurs due to time constraints in overburdened hospitals. Poor communication concerning health risk and the importance of postpartum follow‐up visits and limited consideration of patients' lack of understanding of the issue contribute to poor postnatal follow‐up.^21^, ^22^, ^24^ Low screening uptake is concerning, as crucial early opportunities may be missed. In low‐ and middle‐income countries (LMICs), postpartum screening is largely unavailable due to weak health systems, lack of awareness, difficulty accessing health care, and medications.^25^, ^26^

**TABLE 2:** Factors contributing to poor postpartum follow‐up

Healthcare worker factors	People, family, community factors
Uncertainty about screening recommendations Lack of communication and acceptance of responsibility between obstetrician and primary care provider for ordering screening test Lack of continued vigilance beyond immediate postpartum visit Office location and/or type (hospital‐based or hospital‐affiliated community clinic) Degree of provider specialization Lack of funded national screening programs Inadequate training and tools to provide practical guidance	Low awareness of risk Difficulty to keep screening visit appointment(s) Inadequate family support Lack of prioritization or inadequate attention to women's health Education and financial independence
Barriers	Enablers
OGTT test Environmental context and resources Social influences Emotional aspects Lack of information Memory/attention Social roles Optimism Reinforcement	Knowledge and beliefs about consequences Empowerment for self‐care with practical guidance (not just what to but also how to) Setting goals Behavioral regulation

Current recommendations for postpartum follow‐up include a 2‐hour oral glucose tolerance test (OGTT) at 6–12 weeks postpartum using the diabetes criteria applicable to nonpregnant women, a fasting glucose at 6–13 weeks postpartum, or a glycated hemoglobin (HbA1c) test.^2^ The OGTT is currently considered the gold standard for the detection of diabetes. However, this test is time consuming and requires fasting and multiple blood draws, which often decreases patient compliance, especially for nursing mothers who must accommodate the needs of a newborn. In addition to long waiting times due to high patient load and the 2‐hour test duration, most women from rural communities must travel long distances to receive clinical health care, making the 2‐hour OGTT cumbersome and impractical.^10^, ^21^, ^22^, ^23^, ^24^, ^25^, ^26^

The HbA1c test could alleviate these issues, but it is costly and unaffordable in many LMIC settings. Thus, there is significant impetus for operational research to develop the best protocols based on local conditions, and a need for flexibility of testing these protocols. It is important to emphasize the value of postpartum screening with cultural adaptations to achieve the same in different populations.

### 1.3 FIGO guidance

Considering the increasing global rates of type 2 diabetes and CVD in women, it is essential to address the issue of pregnancy as an opportunity to identify women at risk and provide interventions to improve long‐term health outcomes. The authors of the present article reviewed existing clinical guidelines for postpartum identification and management of women with GDM and identified key recommendations for global clinical practice. The Committee emphasizes that postpartum management of GDM should be considered in the context of a life‐course approach, linking with preconception, postpartum, and interconception services to prevent the development of type 2 diabetes and CVD. This guidance also outlines potential actions to address the barriers to effective communication of risks related to GDM.

### 1.4 Target audience

**FIGURE 1:**
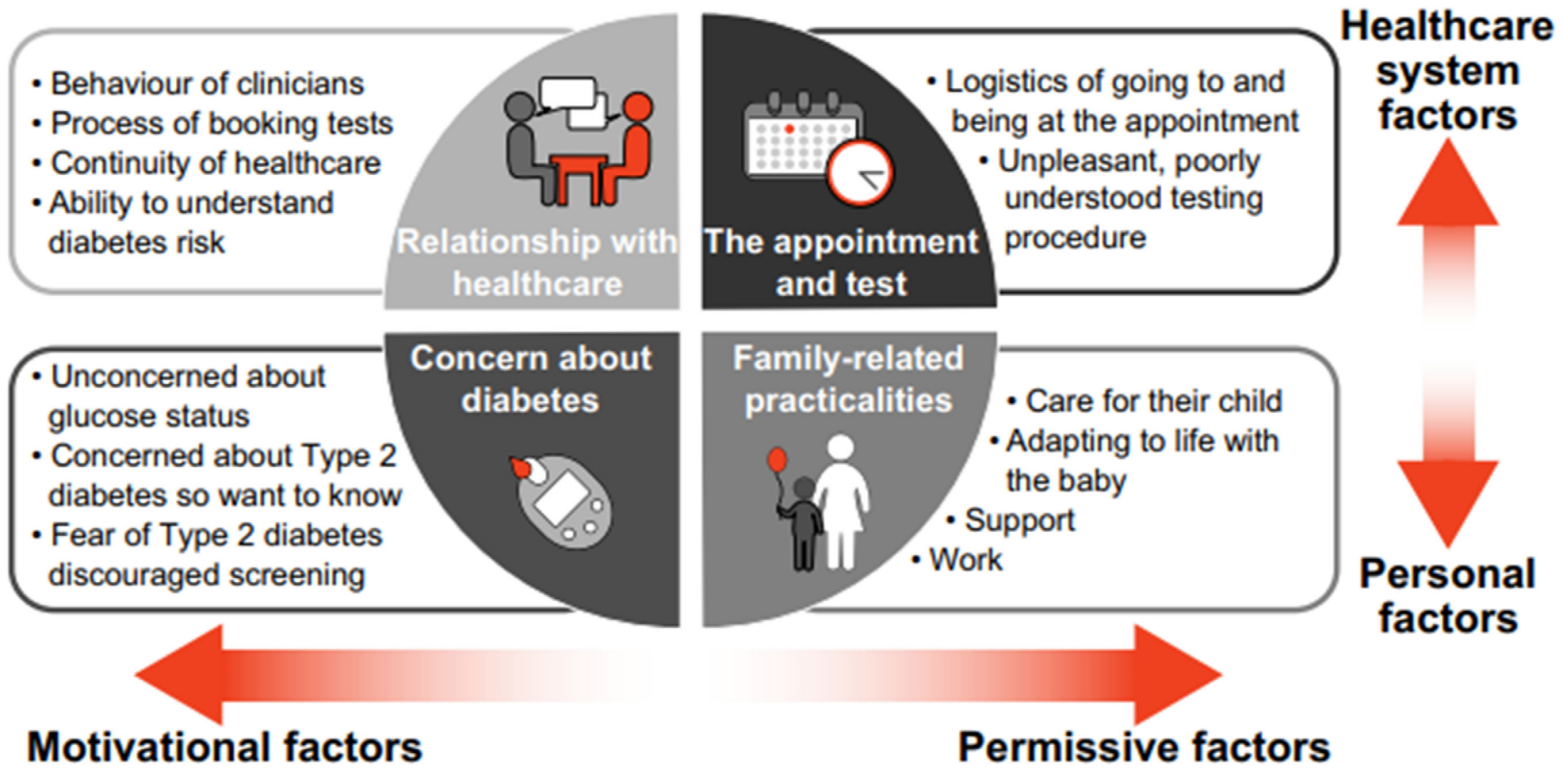
Factors influencing attendance at postpartum follow‐up for glucose testing after GDM. Reproduced from Dennison et al.^27^ under CC BY 4.0 license (http://creativecommons.org/licenses/by/4.0/).

**FIGURE 2:**
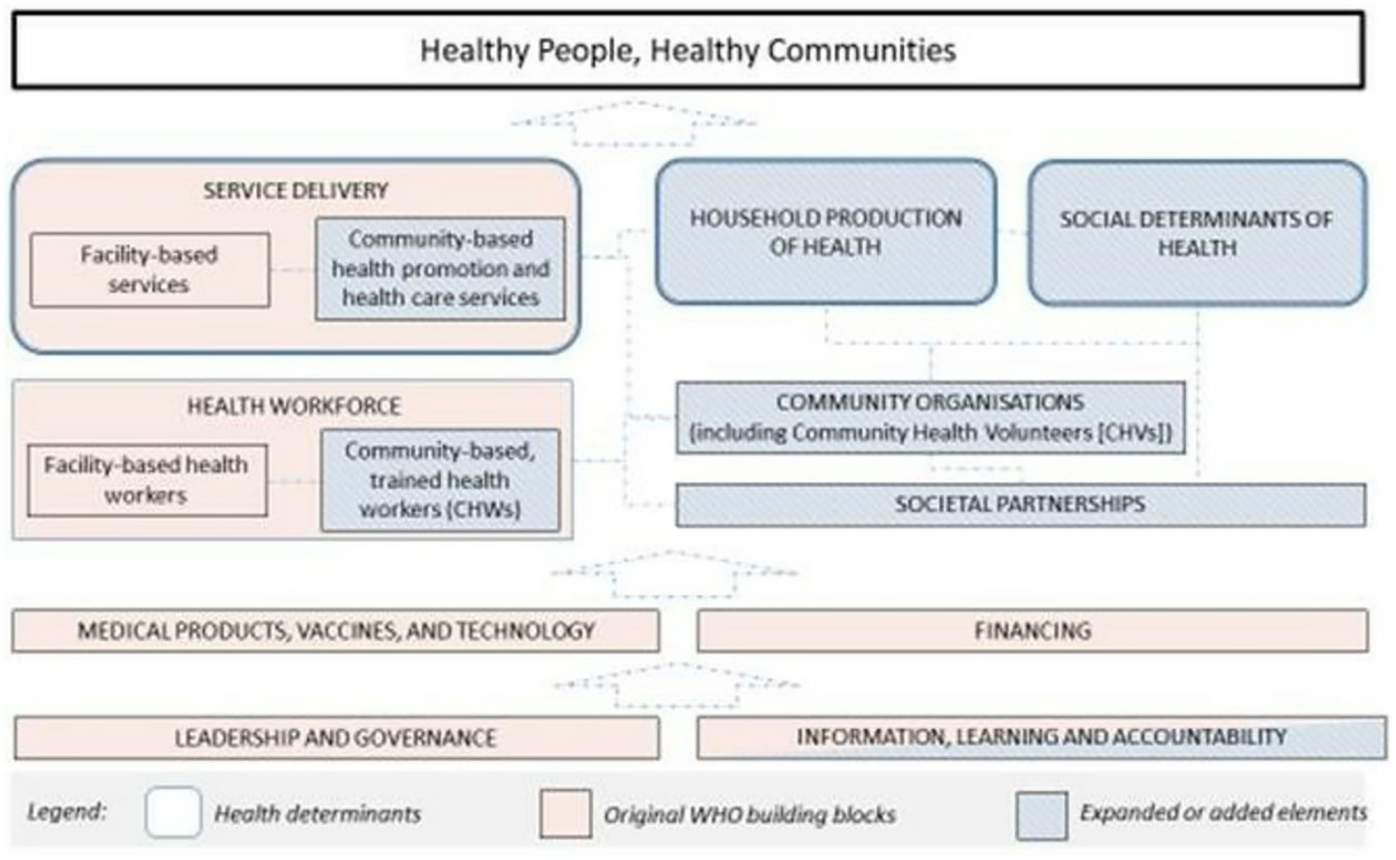
The healthcare system that cares for women with gestational diabetes. Reproduced from Sacks et al.^28^ under CC BY‐NC 4.0 license (http://creativecommons.org/licenses/by‐nc/4.0/).

This guidance is directed at healthcare providers working with women with GDM during and after pregnancy. Management optimally requires a multidisciplinary approach with involvement of a variety of professionals such as general practitioners/family physicians, midwives, nurses, community health workers, dietitians, and nutritionists (Figure 1).^27^ The noncommunicable disease (NCD) prevention guidance outlined here is relevant to individual practitioners providing primary care, gynecological care, and support to women during pregnancy and outside of pregnancy, and their respective professional organizations. This guidance is also relevant to healthcare delivery organizations and providers as it may provide insights into the resource requirements for this group. The healthcare delivery system should consider the revised seven building blocks of the health system as described by the World Health Organization (Figure 2).^28^

## 2 RISK STRATIFICATION OF GESTATIONAL DIABETES AND SUBSEQUENT RISK OF TYPE 2 DIABETES

The risk stratification of women with GDM for subsequent progression to type 2 diabetes can utilize two different approaches: (1) identify additional risk factors specific to the pregnancy complicated by GDM that identify women at higher risk of progression to type 2 diabetes; or (2) evaluate common postpartum factors, with or without consideration of factors present during pregnancy, such as the presence of impaired glucose tolerance (IGT) and/or impaired fasting glucose, or the presence of other established risk factors for incident diabetes. A few examples of the different approaches are discussed here.

Individuals who present with hyperglycemia first diagnosed in pregnancy, during early pregnancy, are more likely to have pre‐existing prediabetes or undiagnosed type 2 diabetes.^2^ These women are, by implication, also at high risk of developing type 2 diabetes postpartum. This is particularly relevant, given the current epidemic of childhood obesity and young‐onset diabetes.

Both maternal body mass index (BMI) and gestational weight gain are important predictors for GDM and are also linked to postpartum weight retention. Women with GDM who are overweight and who subsequently had an increase in postpartum weight have the highest risk of type 2 diabetes progression after delivery.^16^ Even for women with normoglycemia during pregnancy, postpartum weight gain still represents an important risk driver for type 2 diabetes progression, highlighting the importance of postpartum weight management.

Over the past two decades, several diabetes risk models have been developed. These risk models are applicable for the general population and can help to identify individuals with undiagnosed conditions or prioritize individuals with higher risk of type 2 diabetes progression to target them for lifestyle or behavior modification. Such prevention efforts targeting high‐risk individuals are thought to be more cost‐effective than population‐wide intervention efforts,^29^, ^30^ and may help to reduce subsequent type 2 diabetes progression. While pregnancy provides a unique opportunity for healthcare engagement, risk stratification models specifically targeting postpartum women or women with a history of GDM remain scarce. In general, most of the prediction models integrate the major risk factors, such as age, BMI, waist circumference, hypertension, family history of diabetes, and fasting glucose level (Table 3). However, only a few include important predictors such as prior GDM, use of insulin for GDM, and gestational weight gain that are specific for pregnancy, and duration of breastfeeding that is particularly relevant for women postpartum. Thus, there are ongoing efforts to develop specific prediction models for diabetes after pregnancy.^31^

There is increasing interest in the identification of biomarkers that may facilitate stratification of subsequent type 2 diabetes risk among women with GDM. These include genetic variants or polygenic risk scores,^31^ epigenetic markers in blood,^32^ and metabolomics.^33^ While these show promise as potential tools that can further enhance the risk stratification and identification of women most at risk, they are currently limited by their suboptimal sensitivity and specificity, potential costs, and accessibility. The key to implementation of postpartum screening is the need for a pragmatic approach, incorporating relevant simple risk scores or fasting glucose/HbA1c to facilitate uptake and compliance.^29^, ^34^

**Table:** 

**Best practice advice**
Identify women with GDM who are at high risk of progression to type 2 diabetes who require yearly glucose screening: Hyperglycemia diagnosed in the first trimester The degree of glucose intolerance and insulin required during pregnancy Gestational age at diagnosis of GDM Excessive weight gain during pregnancy Inability to return to prepregnancy weight postpartum Recurrence of GDM Absent or short duration of breastfeeding

## 3 POSTPREGNANCY SCREENING

Postpartum testing for ongoing prediabetes and diabetes after an index GDM pregnancy remains challenging. There is inherent conflict between the desire to detect all women at risk and the need for a practical testing regimen that will be implemented into routine clinical practice, rather than simply being recommended in international or national guidelines, but never being undertaken.

A recent study by Balaji et al.^24^ summarized the varying recommendations made by professional bodies. Most of these continue to advocate a formal OGTT at around 6–12 weeks postpartum (up to 6 months according to Diabetes Canada^35^), although the American College of Obstetricians and Gynecologists (ACOG)^36^ and the UK‐based National Institute for Health and Care Excellence (NICE)^37^ promote fasting glucose as the preferred option. Thus, most professional bodies appear to promote “optimal care” using the OGTT. However, due to the cumbersome nature of the OGTT, other studies have sought to evaluate alternative protocols and less onerous tests as alternatives.^38^, ^39^ Waters et al.^40^ evaluated OGTT testing during the immediate postpartum hospitalization period and the conventional 4‐ to 12‐week postpartum timepoint and noted that although a normal early OGTT was able to essentially exclude overt type 2 diabetes at 4–12 weeks (98% negative predictive value/NPV), the NPV for diabetes or prediabetes together was only 75%.

HbA1c appears theoretically attractive as a postpartum test as there is no need for fasting. However, a systematic review^41^ reported a low sensitivity (36%) and moderate specificity (85%) for diabetes using HbA1c and concluded that it was not a suitable test for postpartum diagnosis among women with GDM. Similarly, evaluation of fasting glucose was reported to have a low sensitivity (29%) for diabetes.^42^ However, using a combined strategy including using both HbA1c and fasting glucose to determine the need to proceed to a formal OGTT showed high sensitivity (82%) and specificity (92%), and avoided 70% of the OGTT burden.^43^ Despite a similar report from Picón et al.^44^ the combination of HbA1c and fasting glucose has not been incorporated into any recent guidelines for postpartum testing.

**TABLE 3:** Major risk factors for developing type 2 diabetes after GDM

General factors	Pregnancy‐related factors
Age BMI Waist circumference Hypertension Family history of diabetes Fasting glucose level	GDM Use of insulin for GDM Gestational weight gain Short duration of breastfeeding

Without specific follow‐up and reminder programs, the rates of early postpartum testing following GDM are less than 50%.^45^ On a health system level, lack of clear ‘ownership’ of postpartum care has been identified as a major obstacle.^46^ Transfer of information regarding GDM diagnosis from obstetric/midwifery care during pregnancy to primary care is poor; thus, primary care providers may not have the necessary information to provide appropriate follow‐up strategies. In certain community, primary, or specialist settings, postpartum visits are not routine, which further adds to the problem. Therefore, policy makers and healthcare systems should be strongly encouraged to prioritize systematic postpartum visits for all women with a history of GDM, and the responsibility of postpartum follow‐up bookings and reminders should be allocated to sectors in the healthcare system that are best suited in terms of capability and capacity to undertake this role. Furthermore, a multifaceted strategy incorporating development of capability, opportunity, and motivation should be included to attempt to improve postpartum follow‐up.^47^

Another important issue to consider is the purpose of follow‐up after GDM. If the primary purpose is to allow risk stratification for future diabetes in the immediate postpartum period, then the OGTT, despite its cumbersome nature, may still be the most appropriate test. If an individual woman with prior GDM is planning another pregnancy, then it is arguably worthwhile to accurately diagnose her glycemic status prior to the next pregnancy, and postpartum testing can form part of preconception care. In this situation, accurate detection of IGT or type 2 diabetes is of clear clinical value and may facilitate prepregnancy intervention designed to enhance the outcomes of a subsequent pregnancy. However, from a life‐course perspective, a GDM diagnosis carries substantial long‐term risks, including the development of prediabetes, type 2 diabetes, and CVD.^48^ Although HbA1c testing is inadequate as the sole diagnostic test for postpartum follow‐up, it does represent a viable option for long‐term surveillance, and yearly HbA1c testing has the potential as a diagnostic test for women with a history of GDM in middle‐ to high‐income countries. In addition, measuring the fasting lipid profile (and ideally fasting glucose) to allow early detection of hyperlipidemia would be beneficial.

In a coordinated approach to early detection of vascular risk, a clinical visit at this time should also include measurement of blood pressure, BMI, and abdominal circumference and provide an opportunity for health promotion and education including advice on healthy dietary intake and physical activity. To encourage adherence, such visits could also be integrated with infant care and health promotion including regular vaccinations and child health checks.

**Table:** 

**Best practice advice**
Recommendations for postpartum follow‐up of all women with GDM: Two‐hour oral glucose tolerance test (OGTT) at 6–12 weeks postpartum using the diabetes criteria applicable to nonpregnant women (gold standard) Include a fasting lipid profile and measurement of blood pressure, BMI, and abdominal circumference
**Pragmatic advice**
If best practice cannot be achieved, consider: Fasting glucose at 6–12 weeks postpartum, OR Glycated hemoglobin (HbA1c) Consider combining fasting glucose and HbA1c

## 4 INTERVENTIONS

The low uptake of type 2 diabetes screening after a pregnancy complicated by GDM is well documented.^24^, ^25^, ^26^, ^27^ Interventions to increase screening are needed at personal, practice, and policy levels.

### 4.1 Individual‐level approach

**FIGURE 3:**
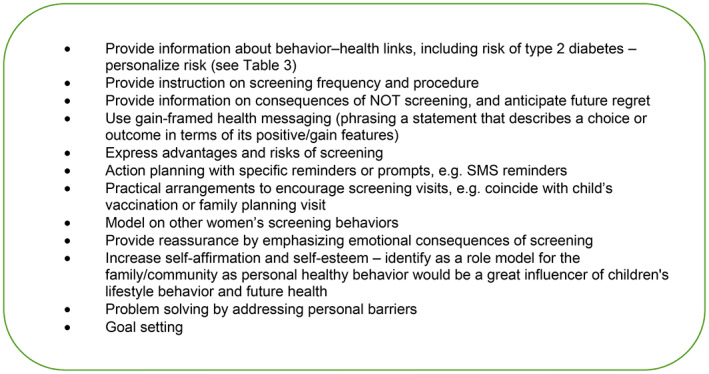
Information to be shared to promote behavior change.

Recommendations on an individual level have focused on increasing knowledge and risk perception among women with GDM, while recommendations to address personal barriers, such as fear of diabetes diagnosis, low prioritization of personal health, and fatalism are still lacking. Enablers at the personal level include social support and feelings of reassurance after screening and family history of diabetes. Women with a family history of diabetes are more likely to be concerned about developing diabetes than women at average risk. These women will also be more likely to adhere to lifestyle modifications to prevent disease.^49^ Thus, personal‐level intervention needs to address a wide range of modifiable factors. Figure 3 outlines the information that should be shared with women to promote behavior change.

#### Lifestyle interventions

Lifestyle modifications such as nutrition, physical activity, and weight loss in women with GDM have been successful in decreasing the progression to type 2 diabetes in several populations, supporting their feasibility as interventions for women with a history of GDM.^6^ Greater weight loss has been observed in overweight women who received individualized diet and physical activity recommendations, diarized daily food intake and activities, and participated in group education sessions compared with the placebo group who received routine care.^50^ When counselling women on lifestyle modifications, support should be provided not just on what to do, but also on how to achieve their goals.^51^

#### Breastfeeding

Breastfeeding is associated with reduced blood glucose levels and incidence of type 2 diabetes among both women with a history of GDM and women in the general population. Breastfeeding has also been associated with postpartum weight loss, reduced long‐term obesity risk, and a lower prevalence of metabolic syndrome.^52^ Stuebe et al.^53^ reported an inverse association between the duration of breastfeeding and type 2 diabetes risk among parous women in the Nurses' Health Study I and II. Among women who had given birth in the previous 15 years, there was a 15% decrease in the risk of type 2 diabetes for each year of lactation, even after adjusting for family history of diabetes, diet, physical activity, and BMI.^54^ To allay concerns that exercise and diet might compromise breastmilk quality, several studies have assessed growth among breastfed infants whose mothers were trying to lose weight and showed no changes in infant weight or length trajectory.^55^ These findings suggest that promotion of a combination of breastfeeding, diet, and physical activity could diminish maternal type 2 diabetes risk without compromising infant growth and might be particularly important in women with a history of GDM. Breastfeeding can be more challenging among women with higher BMI and they may require additional lactation support.^56^

#### Pharmacological interventions

Several randomized clinical trials have studied diabetes prevention with a pharmacologic intervention in women with a history of GDM.^51^, ^56^, ^57^, ^58^, ^59^, ^60^ Troglitazone^59^ and pioglitazone^60^ have been shown to be more effective than placebo in preventing progression to type 2 diabetes but are not recommended for women of reproductive age. Women with a history of GDM (albeit self‐reported) who enrolled in the Diabetes Prevention Program had an impressive response to treatment with metformin, which resulted in a 50% reduction in diabetes risk. In the same study, however, treatment with metformin was associated with only a 14% diabetes risk reduction in women without a previous self‐reported history of GDM.^61^ Although the results of these studies are promising, these medications are not currently recommended for use in diabetes prevention. Further studies are needed to evaluate the relative efficacy and cost of these medications alone or in combination for the prevention of type 2 diabetes in women with a history of GDM.

#### Surgical interventions

Bariatric surgery, where appropriate and available, in appropriately selected obese patients, can reverse glucose intolerance and type 2 diabetes. Nevertheless, women who undergo bariatric surgery should avoid pregnancy until their body weight has plateaued.^62^, ^63^

**Table:** 

**Best practice advice**
Educate on lifestyle interventions such as weight loss via nutritional and exercise interventions; use individualized plans with practical guidance if possible Improve access to lifestyle changes Create environmental opportunities for physical activity, such as safe parks and open spaces Improve access to affordable healthy food outlets Adapt local diets and recipes to be more appropriate for diabetes prevention Encourage breastfeeding for at least 6 months

### 4.2 Population‐level strategies

Policy‐level factors include screening type and requirements, coordinated reminder systems, and protocols for recording and communicating GDM history. Population‐level interventions are needed to increase awareness and acceptance of the risk for the development of type 2 diabetes in women with a history of GDM. Public education campaigns and online information about susceptibility to type 2 diabetes, screening frequency, health consequences of not screening, and its effects on future pregnancies could increase awareness of patient risk. Furthermore, discussing management strategies, if and when diabetes is diagnosed, offers reassurance of health when diabetes status is known, and acknowledging negative feelings will encourage focus on achieving the screening goal. Policies should also be aimed at improving access and accommodating lifestyle changes. Moreover, population‐level strategies should focus on the role of women's health in society and highlight the importance of postpregnancy screening and care, balancing the role of mother and self‐care, and screening for gender‐based violence and mental health issues.^64^, ^65^

### 4.3 Implementation

From the health system perspective, absence of standardized postpartum care for women has been identified as a barrier. Specifically, diabetes‐related policies meant for doctors do not reach the primary care clinic. This gap in communication between the delivery unit where women deliver and the primary care clinic where they return for postpartum check‐ups has led to confusion and uncertainty among healthcare providers regarding postpartum screening for diabetes.^21^, ^22^, ^23^, ^24^, ^25^, ^26^, ^27^, ^28^

The rate of adherence to postpartum follow‐up is improved when proactive systems are in place, in addition to routine care. Providing an option for home blood sample collection for completing the OGTT has been shown to increase the rate of follow‐up.^24^, ^66^, ^67^ Moreover, evidence suggests that sending reminders to patients to return for follow‐up by telephone, e‐mail, or SMS increased the odds of a postpartum visit three‐fold compared with no reminders.^68^ However, a personalized approach, such as making telephone calls in lieu of emails or letters, has been shown to improve screening rates and enhance patient commitment. Further assessment demonstrated that women who participated in the trial preferred SMS over email, letters, or voice calls.^68^

Barriers and factors contributing to poor postpartum screening at both individual and healthcare provider level must be addressed to improve compliance rates and ensure that women with a history of GDM do not undergo subsequent pregnancies with undetected diabetes. There are several ways in which the healthcare system can address the low postpartum follow‐up rates and improve compliance. These include: (1) staying updated on current recommendations; (2) providing dedicated teams of professionals to provide personalized counselling; (3) implementing home care services where necessary; (4) introducing proactive systems such as recall registries; (5) linking postpartum care to the offspring's vaccination and baby care program; (6) using reminder systems; and (7) providing women adequate information about the impact of GDM on their health. After delivery, women regularly visit their family doctor or pediatrician for child vaccination and development milestones. This could be an important opportunity to remind women of their risk and motivate them to undergo regular testing. In this regard, family physicians should be made aware of a woman's history of GDM.^22^, ^23^, ^24^, ^26^

According to medical providers, two of the most difficult balances when communicating the diagnosis of GDM to pregnant women are: (1) communicating risks but still providing reassurance; and (2) addressing immediate dangers to the infant without minimizing future risks to the mother. The complex messages that GDM entails and a multimember clinical team without a manager accentuate the prevailing confusion among patients as they try to understand and navigate GDM. No individual team member has sufficient knowledge or time to independently manage the totality of patient care, and yet no individual is designated to direct or coordinate the care. Several important questions need to be considered. These questions include: Who should take responsibility for arranging a test? When should the test be arranged? Whose responsibility it is to issue a follow‐up reminder or report results when the patient has been appropriately discharged prior to the arranged test? Both providers and patients have little time and motivation to perform or receive postpartum testing, and thus miss the opportunity to launch ongoing monitoring and life‐course prevention. It is, therefore, imperative that every member of the healthcare system reinforces the importance of postpartum screening, and that in both primary and secondary care, proper communication channels are set up to ensure that women with GDM receive postpartum testing and do not undergo subsequent pregnancies with undiagnosed diabetes. These interventions among physicians could improve the chances for healthy pregnancies among women with a history of GDM.

The use of mobile health (mHealth) technology—specifically mobile applications on smartphones to deliver individual‐level care above traditional clinic‐based care—provides a unique opportunity to communicate with and motivate women during pregnancy and to return for postpartum follow‐up.^69^, ^70^ Results from randomized controlled trials involving mobile phone application‐based interventions, biosensor/activity monitors (pedometers), web‐based dietary intervention, interactive communication between participant and healthcare professionals, and bluetooth‐enabled glucometers show promising results for self‐management of diabetes. A recent review consolidating evidence from several systematic reviews on the effectiveness of mHealth interventions for patients with diabetes concluded that mHealth interventions represent a promising approach for self‐managing diabetes and weight management.^71^

While longer‐term engagement poses many challenges, it must be noted that preventive activities have a legacy effect and thus even a year of moderate intensity intervention postpartum has great potential in delaying the onset of or preventing type 2 diabetes.

Low‐resource contexts pose a challenge^20^; for example, the model of care in which women with GDM were followed up throughout their pregnancy by trained healthcare professionals was tested in a low‐resource setting.^72^ This model included face‐to‐face counselling with nutritionists and healthcare professionals, whereby women were educated about GDM and its adverse health effects on both mother and baby, were provided with educational booklets, and were motivated to track their dietary pattern and physical activity. The model was found to be effective in reducing the rate of both maternal and neonatal complications in women with GDM to levels similar to women with normoglycemia.^5^ After delivery, women were followed up via telephone calls and were reminded to return for postpartum testing. For women who were unable to return to the hospital, postpartum testing was arranged for them at home. In several parts of Asia and Africa many women move from their place of residence to relatives for delivery. Therefore, demographic and contact details of the women's family were collected during the study so that relatives could be contacted if necessary. Women who were not expected to return shortly after the delivery were requested to undergo postpartum testing for diabetes in a hospital or laboratory close to their home. Women who had left the country after delivery were contacted through WhatsApp and email and reminded about undergoing testing, to which these women complied and sent back their OGTT results. Using this model of care, a 95.8% postpartum follow‐up rate was achieved, proving to be successful in a low‐resource setting.^73^

**Table:** 

**Best practice advice**
Improve postpartum follow‐up rates by: Coordinated postnatal care supported by a case manager or healthcare worker, reduced waiting times, clear communication about health, practical guidance on nutrition and lifestyle, vaccination, and baby care programs Using reminder systems Implementing and encouraging home/self‐testing

## 5 OPPORTUNITIES FOR FUTURE FOLLOW‐UP AND INTERVENTION

The postpartum period provides a window of opportunity to prepare for healthy future pregnancies and to identify women with a higher risk of type 2 diabetes. This period also allows for an opportunity to address contraceptive needs, reversal of excess gestational weight gain, discussion of nutritional requirements, and screening for postpartum depression. If the pathways toward chronic illnesses anticipated by pregnancy complications are to be interrupted and subsequent disease prevented, healthcare practices and systems need to be able to respond to the warning signals in pregnancy. However, most women at risk do not visit a primary care provider in the year after giving birth.

Considering a mother's physical and mental health in the postpartum period, several experts have recognized the importance of providing continuity of care in the postpartum period—known as the ‘fourth trimester’. Key service providers for infant health such as pediatricians, general practitioners, and health visitors have an important role in discussing interpregnancy health, not only for the next pregnancy but also for the long‐term health of the mother. Not addressing these issues before the next pregnancy is a missed opportunity for improving women's health and outcomes of subsequent pregnancies. FIGO recommends the extension of preconception care into the postpartum stage to increase the window of opportunity and to access women with additional needs, thus providing an integrated continuum of care for women.

Six themes of interacting influences on postpartum behavior have been identified and require further exploration: (1) role as mother and priorities; (2) social support; (3) demands of life; (4) personal preferences and experiences; (5) risk perception and information; and (6) finances and resources (plus preferred format of interventions). These factors prevented many women from addressing their own health, while they motivated others to persevere. Adherence to postpartum follow‐up can be improved if diabetes screening is linked to child vaccination programs or family planning visits, and with the use of mHealth and virtual visits.

## 6 CURRENT CHALLENGES AND RESEARCH OPPORTUNITIES

The prevalence of both GDM and type 2 diabetes is increasing. If a woman is diagnosed with GDM, she is identified as having an increased risk of type 2 diabetes. However, the intensity and duration of preventive actions for these women has not been optimized. Future research should focus on:

- Improving preconceptual/interpregnancy care
- Maternal nutrition and reducing obesity
- Identification of novel biomarkers and cost‐effective tests for diabetes
- Implementation and impact of recommendations in real‐world settings

**FIGURE 4:**
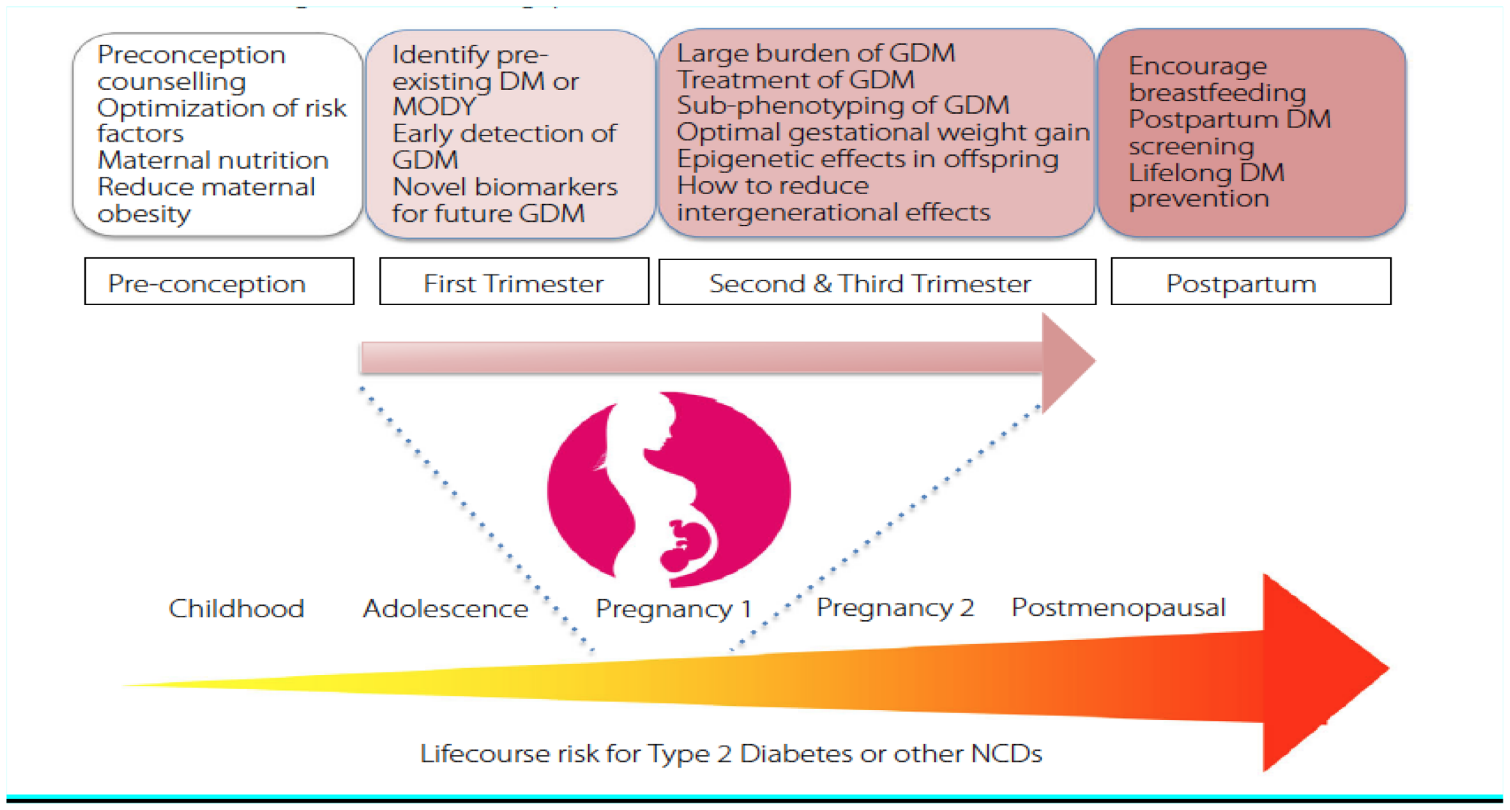
Current challenges and research gaps in gestational diabetes mellitus. Reproduced from Chu et al.^74^ under CC BY‐NC‐ND license (https://creativecommons.org/licenses/by‐nc‐nd/4.0/).

Figure 4 summarizes the current challenges and research priority areas that can be tackled throughout a woman's reproductive life.^74^

## AUTHOR CONTRIBUTIONS

SA, HDM, AK, RCM, and FMA conceptualized the article. SA, HDM, SD, KYT, and RCM compiled the article with contribution from all authors. FMA was responsible for final editing.

## CONFLICT OF INTEREST

Harold David McIntyre reports honoraria for lectures from Phillips Health Care, Mead Johnson (China), and Diabetes Ireland. Ronald Ma reports research support from AstraZeneca, Bayer, Novo Nordisk, Pfizer, and Tricida Inc. Lina Bergman reports research funds from Thermo Fischer, Roche, and Perkin Elmer and payment from Homburg and Partner. Cynthia Maxwell reports grants from the Canadian Institutes for Health Research and the Crohns and Colitis Foundation of Canada. Sharleen O'Reilly reports research grants from the European Commission Horizon 2020, National Health and Medical Research Council of Australia, Health Research Board Ireland, Al Qasimi Foundation, and University of Sharjah. Other authors have no conflicts of interests to declare.
